# Nutritional Pearls and Pitfalls of Gastrointestinal Diseases

**DOI:** 10.3390/nu15132889

**Published:** 2023-06-26

**Authors:** Massimo Bellini, Christian Lambiase

**Affiliations:** Gastrointestinal Unit, Department of Translational Research and New Technologies in Medicine and Surgery, University of Pisa, 56124 Pisa, Italy

Since ancient times, food has been considered a possible therapy for treating a wide range of diseases. Many people view food as the primary source of their health, and a nutritional approach is frequently thought to be safer than conventional pharmacological therapy because it is assumed that anything that is “natural” is either beneficial or harmless. Therefore, the nutritional approach in managing gastrointestinal diseases has recently become increasingly popular among both patients and healthcare professionals.

People frequently tend to overestimate the benefits of certain food-based substances and/or underestimate the capacity of foods to interact metabolically with the drugs they are already taking. Moreover, many patients start diets, even the most odd and bizarre, following the suggestions of friends and relatives, without consultation with a healthcare professional or expert supervision. Furthermore, messages sent by the media regarding these topics are frequently distorted.

Based on the above background, the aims of this Special Issue were to analyze the actual facts and the false beliefs regarding the possible positive and negative impacts of food and, in general, of the dietary approach for many different gastrointestinal diseases.

This Special Issue entitled “Nutritional Pearls and Pitfalls of Gastrointestinal Diseases”, comprises 16 peer-reviewed papers reporting on several clinical and nutritional aspects related to food in the management of a large variety of gastrointestinal diseases. These include disorders of gut–brain interactions (DGBI), inflammatory bowel diseases (IBD), liver pathology, diverticular disease, eosinophilic esophagitis (EoE), allergies and intolerances, and nutritional approaches in pediatric and elderly patients. 

Nutritional approaches may start from the pediatric age, as discussed by Bivi et al. [1] and Di Chio et al. [2] in their papers. Bivi et al. [1] carried out a national cross-sectional survey involving parents of children following a vegan diet in order to explore the difficulties perceived by parents in their relationship with primary care pediatricians (PCPs) regarding a vegan diet. Interestingly, the results of this study showed that more than 1/3 of parents did not inform their PCPs about the vegan diet, mainly because PCPs were perceived as skeptical or against a vegan diet [1]. On the other hand, about 70% of the parents relied on medical dietitians, and 28.2% on nutritionists/dietitians for dietary counseling [1]. 

Di Chio et al. [2] focused on aspects regarding pediatric nutritional management in gastrointestinal diseases, giving an overview on advances, “false beliefs”, and the recent evidence in the nutritional approach to pediatric gastrointestinal diseases. The authors emphasized, for all therapies, that dietary changes can be harmful in the pediatric setting. Regarding the role of food, authors highlight that it may be the trigger of specific diseases (e.g., IgE or non-IgE gastrointestinal food allergies, celiac disease, food protein-induced enterocolitis syndrome, or eosinophilic gastrointestinal diseases) and suggest that if a “trigger food” is clearly identified, the specific food elimination diet can be the best therapeutic choice for inducing remission [2].

Neri and d’Alba [3] focused their attention on the pathophysiology of malnutrition in elderly people. This was with the aim of understanding and promoting the knowledge of age-related changes in appetite, food intake, and reduction in homeostatic capacity, physiological functional reserves, and body composition. After a thorough and complete review of the pathophysiological mechanisms leading to malnutrition and sarcopenia in aged people, the authors concluded that recognizing risk factors is mandatory in treating malnutrition in elderly patients. 

Gargano et al. [4] discuss and provide an outline of the pathophysiologic and clinical features of immune and non-immune adverse reactions to food along with general diagnostic and therapeutic strategies. This is an attempt to offer a useful tool to practicing physicians in discriminating these diverging disease entities and planning their correct management [4]. 

Visaggi et al. [5] investigated dietary management strategies for EoE, discussing endpoints, rationale, advantages and disadvantages, and the currently available dietary regimens for EoE. The authors concluded that, among the different dietary strategies, elimination diets represent the most effective treatment for EoE, with a 90.8% efficacy at all ages. However, burdensomeness and high costs make these diets only suitable for the short-term treatment of select patients. Furthermore, the authors discourage performing skin prick, atopy patch, and food-specific IgE tests to guide dietary restrictions due to a lack of reproducibility and low predictive value.

The paper by Usai-Satta et al. [6] discusses the diagnostic role of hydrogen breath testing (BT) in the nutritional management of carbohydrate malabsorption (i.e., lactose malabsorption and intolerance, fructose malabsorption, sorbitol malabsorption, and FODMAP intolerance). The authors concluded that lactose BT can be recommended in clinical practice in patients with abdominal pain, bloating, and diarrhea. Unlike lactose, BTs for other sugars, such as fructose and sorbitol, add no diagnostic advantage to guiding the nutritional management of these intolerances compared with direct dietary intervention.

Guida et al. [7] and Bertani et al. [8] focused on the role of nutritional approaches in patients with inflammatory bowel disease (IBD). Guida et al. [7] investigated, through a survey, the food habits and nutritional knowledge of a homogeneous cohort of patients with IBD from southern Italy. They concluded that most IBD patients begin diets and undergo lifestyle changes on the basis of self-experience and give up many foods that are considered triggers for their symptoms. This can lead to psychosocial consequences and nutritional deficiencies. 

In their review Bertani et al. [8] explored the current knowledge regarding nutritional recommendations in IBD. The authors highlight that Westernized dietary patterns are a risk factor for IBD, through the promotion of a dysbiotic profile and the production of intestinal inflammation. Furthermore, in active disease, the authors strongly recommend an accurate assessment of the patients’ nutritional status and suggest an early treatment of protein/energy malnutrition and micronutrient/vitamin deficiencies. 

Mega et al. [9] conducted a review regarding the role of food and nutrition on the pathophysiological mechanisms leading to liver injury. They highlight the fact that some foods are involved in the pathogenesis of liver damage while others have been recognized to have a protective effect in several liver diseases. Plant-derived foods (especially cereals) are more protective against chronic liver disease than animal-derived foods. Some animal food products (red or processed meats) tend to increase the risk of liver damage. In this context, the authors underline the strong recommendation for a “Mediterranean diet” to decrease the incidence of cardiovascular disease, metabolic syndrome, and type 2 diabetes. 

In their paper, Furbetta et al. [10] focused on the role of perioperative nutritional aspects in total pancreasectomy. The results presented in this review suggest that a preoperative assessment of nutritional status is recommended because these are prognostic factors in both total pancreasectomy for pancreatic cancer to support chemotherapy and in total pancreasectomy with islet auto-transplantation for chronic pancreatitis. The authors stress that enteral nutrition is always preferable to parenteral nutrition in these patients. 

The systematic review conducted by Carabotti et al. [11] summarized the evidence available regarding the role of dietary habits in the prevention of diverticular disease complications. A high intake of fiber is associated with a decreased risk of diverticulitis or hospitalization due to diverticular disease, with a protective effect of fruits and cereal fiber alone (but not all vegetable fibers). On the other hand, high red meat consumption and a generally Western dietary pattern are associated with an increased risk of diverticulitis. Furthermore, alcohol intake seems to be linked with diverticular bleeding, but not to recurrent diverticulitis or diverticular complications. 

Finally, several papers focused on the pearls and pitfalls of nutritional approaches in the management of DGBI [12,13,14,15,16].

Duncanson et al. [12] explored the relationship between foods and symptoms in functional dyspepsia (FD), focusing on the possible mechanisms of eating-induced and food-related symptoms and the possible role of nutritional management of FD symptoms. Specific carbohydrates, proteins, and fats, or foods high in these macronutrients have all been reported as influencing FD symptom induction, with removal of “trigger” foods or nutrients shown to alleviate symptoms. Emerging evidence in the literature suggests that the gastrointestinal microbiota might be related to food and symptom induction in FD. 

In his paper, Spiller [13] highlights that both patients and clinicians identify diet as a strong factor associated with IBS symptoms and attempted to detect possible links between specific groups of foods and IBS symptoms. The author suggests that management should start with the NICE guidelines. In the case of failure, it is advisable to start with a trial of a simple exclusion diet (i.e., wheat, milk, excessive caffeine, and/or specific foods that the patient has identified). Further steps may include the referral to a dietician for a trial of a low FODMAP diet.

The two papers by O’Brien focused on the dietary management of patients with chronic diarrhea [14,15]. In the review, O’Brien et al. [14] present the advantages of a suitable dietary approach in improving digestive symptoms, energy levels, reducing reliance or need for pharmaceutical medications, and increasing quality of life. These authors also present the pitfalls of such dietary changes, especially the impact upon the gut microbiota and subsequent overall health of patients. In the second paper, O’Brien et al. [15] explored the positive and negative impacts of a low FODMAP diet in older adults with chronic diarrhea. Twenty over-65 patients with chronic diarrhea were enrolled and underwent a 6-week low FODMAP diet. The authors report a clinically significant improvement in total gastrointestinal symptoms, including diarrhea, and a significant reduction in anxiety.

The narrative review of Bellini et al. [16] aimed to clarify the role of a nutritional approach in managing chronic constipation (CC). The authors concluded that the nutritional approach has an important role in chronic constipation treatment in everyday clinical practice because it is the cornerstone on which CC therapy should be built. The authors also explore the most suitable dietary approach for treating patients based on CC pathophysiology.

In conclusion, a dietary approach may be indicated in many gastrointestinal diseases, both functional and organic, from pediatric to old age. Unfortunately, there is often a lack of specific knowledge regarding this issue and the available therapies and approaches on the part of both patients and physicians. In the near future, it will be mandatory to fill these gaps in order to enhance the pearls that nutritional managements can offer and avoid the dangerous pitfalls. 
